# Current vegetation data from the Prioksko-Terrasnyi Biosphere Reserve

**DOI:** 10.3897/BDJ.9.e71266

**Published:** 2021-08-25

**Authors:** Mikhail Shovkun, Natalya Ivanova, Larisa Khanina, Michael S. Romanov, Vasily Demidov

**Affiliations:** 1 Prioksko-Terrasnyi Biosphere Reserve, Danki, Russia Prioksko-Terrasnyi Biosphere Reserve Danki Russia; 2 Institute of Mathematical Problems of Biology RAS – branch of the Keldysh Institute of Applied Mathematics of Russian Academy of Sciences, Pushchino, Russia Institute of Mathematical Problems of Biology RAS – branch of the Keldysh Institute of Applied Mathematics of Russian Academy of Sciences Pushchino Russia

**Keywords:** Russia, Moscow Region, mixed coniferous forests, forest types, sampling-event data, Darwin Core.

## Abstract

**Background:**

Here we present the sampling event dataset that contributes to the knowledge of current vegetation of the Prioksko-Terrasnyi Biosphere Reserve (part of the UNESCO World Network of Biosphere Reserves), Moscow Region, Russia. The Reserve is situated on the terraces of the Oka River in the zone of mixed coniferous forests.

**New information:**

The dataset provides 269 relevés (9174 associated occurrences) of renewed vegetation collected in 2019-2020. It is aimed at sampling vegetation data from the Reserve area with particular interest to sites with invasive species and sites with recent deadfall in the spruce stands caused by the bark beetle-typographer. The dataset contains representative information on plant communities in localities with assigned GPS coordinates, sampled using the standard relevé method with the Braun-Blanquet cover-abundance scale. During this study, we found two vascular plant species included in the Red Data Book of the Russian Federation, 25 species from the Red Data Book of Moscow Region, as well as 19 alien vascular plant species for the Reserve. These data contribute to our knowledge of species composition and structure of a renewed vegetation of the Reserve, protected and alien species distribution.

## Introduction

The Prioksko-Terrasnyi Biosphere Reserve is located 80 km south of Moscow and is managed by the Federal State Budgetary Institution “Prioksko-Terrasnyi Nature Biosphere Reserve”. The Reserve was established on 19 June 1945; it occupies an area of 4960 hectares. According to the classification of the International Union for Conservation of Nature (IUCN), the Reserve belongs to the Ia category: Strict Nature Reserve (State Nature Reserve). The Reserve manages conservation and restoration of natural ecosystems, landscapes, rare and endangered species of plants and animals; preserves and studies unique meadow-steppe vegetation communities and floristic complexes, known as “the Oka River flora”; carries out long-term comprehensive environmental studies and environmental education. In addition, the Breeding Centre for European Bison has also functioned in the Reserve since 1948. The Reserve has been a part of the UNESCO World Network of Biosphere Reserves since 1979.

Vegetation studies have been carried out in this area even before the Reserve foundation. In 1861, Russian botanist Nikolay Kauffmann made the first flora observations (Kauffmann 1866). He was the first scientist to note the unusual presence of steppe vegetation along the Oka River in the midst of mixed coniferous and broad-leaf forest zone. In the following years, interest amongst botanists to this place increased, which finally led to establishing the Nature Reserve here in 1945. From 1945 to 2021, numerous vegetation studies have been undertaken (Smirnov 1958, Skvortsov 1969, Danilov et al. 1981, Danilov 1983, Levitskaya 1993, Smirnova and Popadyuk 1999, Zaugolnova 1999, Smirnova 2000, Khanina et al. 2000, Smirnov et al. 2001, Zaugolnova and Esipova 2000, Alekseev et al. 2004, Bobrovsky and Khanina 2004, Bobrovsky and Khanina 2005, Bobrovsky and Brynskikh 2005, Denisova and Bronnikova 2005, Popchenko 2011, Zelenskaya 2011, Andreeva and Mikhaleva 2012, Andreeva and Onipchenko 2012, Andreeva and Onipchenko 2015, Zelenskaya and Kerzhentsev 2015 and others). The first complete vegetation mapping of the Reserve was undertaken by the Oka complex expedition in 1949 (Prioksko-Terrasnyi Biosphere Reserve 1949). Revisions to the map and vegetation mapping of specific sites with steppe flora were done in later years (Prioksko-Terrasnyi Biosphere Reserve 1974, Prioksko-Terrasnyi Biosphere Reserve 1984b, Prioksko-Terrasnyi Biosphere Reserve 1984a) along with forest inventory mappings, which were repeated every 10-15 years. In 2015, the map of the “Groups of associations of current vegetation of the Prioksko-Terrasnyi Reserve” was developed as a part of the Reserve’s forest inventory (Prioksko-Terrasnyi Biosphere Reserve 2015).

**Figure 1.**
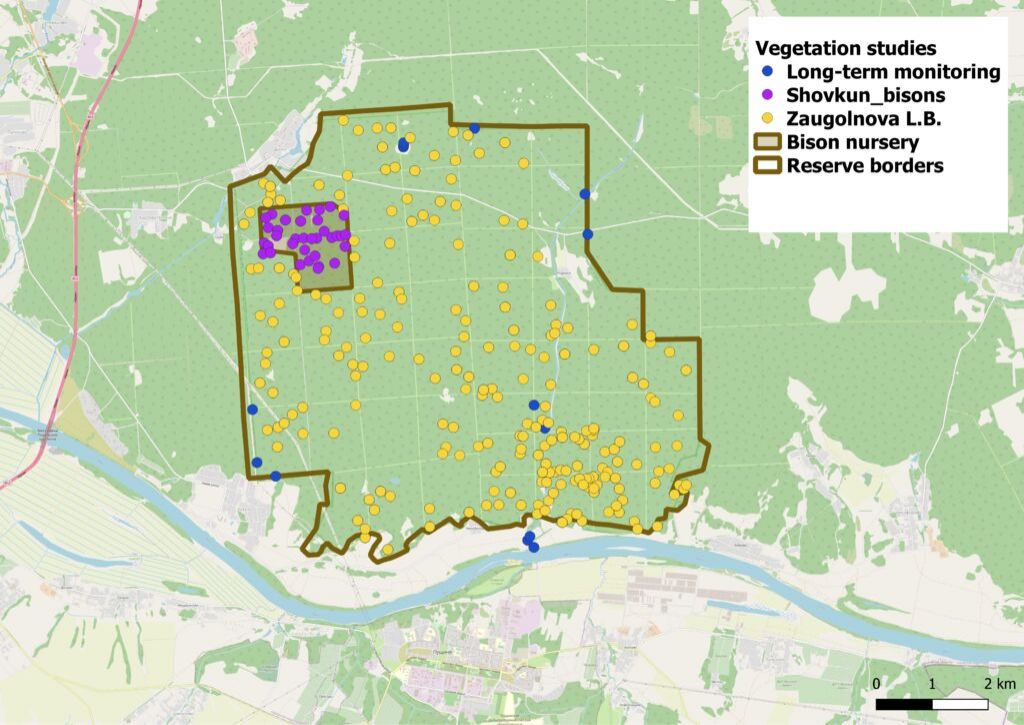
Locations of vegetation plots sampled during the previous studies in the Reserve. Reserve borders from World Resources Institute et al. 2012). Geographic database from OpenStrretMap (OpenStreetMap contributors 2015) via QGIS (QGIS Development Team 2020).

Some of phytosociological data, collected in the Reserve, are already available through the European Vegetation Archive (EVA), which is a repository of vegetation plots from Europe and adjacent areas (Chytrý 2016). The database “Temperate Forests of European Russia” (Khanina and Bobrovsky 2018) includes relevés sampled by a large team under the guidance of Prof. Olga V. Smirnova and Prof. Ludmila B. Zaugolnova in the 1990s, temporary plots sampled in the Bison nursery by Mikhail Shovkun in 2001 (Shovkun 2003) and long-term surveys on permanent sampling plots, collected under the supervision of Prof. Vladimir Onipchenko (since 1991). Locations of these plots are shown in Fig. 1.

**Figure 2.**
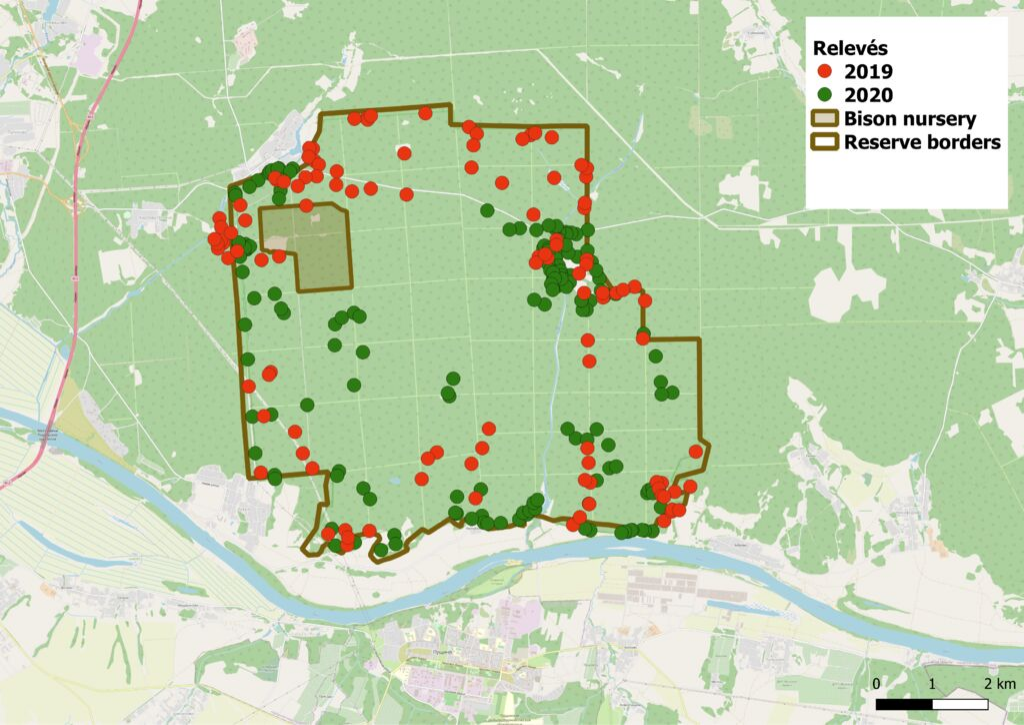
Locations of relevés sampled in 2019 and 2020 in the Reserve. Reserve borders from World Resources Institute et al. 2012). Geographic database from OpenStreetMap (OpenStreetMap contributors 2015) via QGIS (QGIS Development Team 2020).

The presented data of 2019-2020 (Shovkun 2021) provide the results of renewed vegetation studies (Fig. 2). It is aimed at sampling vegetation data from the Reserve area with particular interest to sites with invasive species and sites with recent deadfall in the spruce stands caused by the bark beetle-typographer (*Ips typographus* (C. Linnaeus, 1758)). The vegetation data sampled there will provide insight into the processes of invasions and natural reforestation.

## General description

### Purpose

The importance of the study is due to the fact that it was conducted in the Reserve where all kinds of felling and any other economic activities are prohibited. As a result, the natural ecological succession has not been interrupted by human-beings since 1945. Thus, the data collected in 2019-2020 at the particular stage of forest succession can be used in future studies to assess characteristics of ecological succession and a process of the restoration of natural mixed coniferous - broad-leaved forests in the Reserve area.

## Sampling methods

### Study extent

The Reserve is situated on the ancient terraces of the Oka River in the region of mixed coniferous forests. However, vegetation is now presented by early succession forest communities (dominated by Scots pine and silver and downy birch), developed on sites which have experienced strong anthropogenic impacts in the past: logging, grazing, ploughing, wildfire etc. (Ivanov et al. 2006). The earliest changes to prehistoric ecosystems were probably made during the Bronze Age by slash and burn agriculture, wildfires and grazing. The strongest pressure from human activity can be observed in the 15th-16th centuries when several villages (later abandoned) existed in the area of the future Nature Reserve (Demidov 2019). Intensive logging continued throughout the 18th to 20th centuries, peaking during Word War II from 1941 to 1945. There were some severe fires in the early 20th century. With the Reserve establishment, all logging, grazing and any crossing of the area were strictly prohibited and these rules are still in force today. Since 1945, forest fires have had little or no effect on vegetation.

### Sampling description

In the growing seasons 2019 and 2020, vegetation was surveyed in 269 temporary plots: 111 in 2019 and 158 in 2020. The data were sampled according to the relevé method (plot size was 100 m^2^, 10 × 10 m) using the Braun-Blanquet cover-abundance scale (Mueller-Dombois and Ellenberg 1974, Chytrý and Otýpková 2003). The total cover and individual cover by species for all vascular plants were estimated for the following vegetation layers: the tree canopy layer (the overstorey), the understorey layer including tree undergrowth and tall shrubs and the field layer comprising the herbaceous species, together with tree and shrub seedlings. The position of the centre of each site was georeferenced using GPS receiver in WGS84 datum. Locations of relevés are shown in Fig. 2.

### Quality control

Species were identified using the key (Gubanov et al. 1995) by Mikhail Shovkun. He is a specialist in botany and has a wide experience in floristic studies of this region (Alekseev et al. 2004, Shovkun 2003). Scientific names were checked using the GBIF species matching tool. Geographical coordinates of each relevé were checked according to available forest inventory data.

### Step description

For data analysis, we used the classification of forest types described in Smirnova et al. (2017) (pp. 545–551). The forest types were determined by a combination of tree species dominating in the canopy and an ecological-coenotic species group (ECG) dominating in the ground layer. ECG was earlier defined as a group of species similar in ecological features and in constancy of occurrence in different types of vegetation communities (Nitsenko 1969, Smirnov et al. 2006, Smirnov et al. 2008, Smirnova et al. 2017). In this work, we used seven ECGs: boreal (Br), nemoral (Nm), nitrophilous (Nt), oligotrophic (Olg), pine-forest (Pn), water-marsh (Wt) and meadow-edge (Md). Distinguished forest types are presented in Table 1, as well as being available in the GBIF dataset (Shovkun 2021).

## Geographic coverage

### Description

Moscow region, Russia

### Coordinates

54.85103 and 54.9209 Latitude; 37.5505 and 37.68457 Longitude.

## Taxonomic coverage

### Description

The dataset includes 564 unique scientific names, mainly of phylum Tracheophyta (542 taxa were identified to species and subspecies ranks and one taxon to genus rank only). We also recorded one species and one genus of phylum Marchantiophyta, four species and 15 genera of Bryophyta and one genus of Ascomycota. For these groups, only conspicuous taxa were counted, so these data are not complete. However, we include these occurrences in the dataset because GBIF data on these taxa are very limited.

During vegetation studies we counted occurrences of two vascular plant species included in the Red Data Book of the Russian Federation (Bardunov and Novikov 2008): *Fritillaria ruthenica* Wikstr. and *Neottianthe cucullata* (L.) Schltr. Moreover, we found 25 species from the Red Data Book of Moscow Region (Varlygina et al. 2018): *Melica picta* K.Koch, *Fritillaria ruthenica* Wikstr., *Allium ursinum* L., *Iris sibirica* L., *Neottianthe cucullata* (L.) Schltr., *Platanthera chlorantha* (Custer) Rchb., *Aconitum nemorosum* M.Bieb. ex Rchb., *Clematis recta* L., *Pulsatilla patens* (L.) Mill., *Alyssum gmelinii* Jord. & Fourr., *Jovibarba sobolifera* (J.Sims) Opiz, *Cerasus fruticosa* Pall., *Rosa villosa* L., *Conioselinum tataricum* Hoffm., *Chimaphila umbellata* (L.) W.P.C. Barton, *Gentiana cruciata* L., *Pulmonaria angustifolia* L., *Dracocephalum ruyschiana* L. , *Pedicularis kaufmannii* Pinzger,*Scrophularia umbrosa* Dumort., Crepis praemorsa (L.) Tausch, *Ligularia sibirica* (L.) Cass., *Scorzonera humilis* L., *Serratula coronata* L. and *Veratrum nigrum* L.

**Figure 3.**
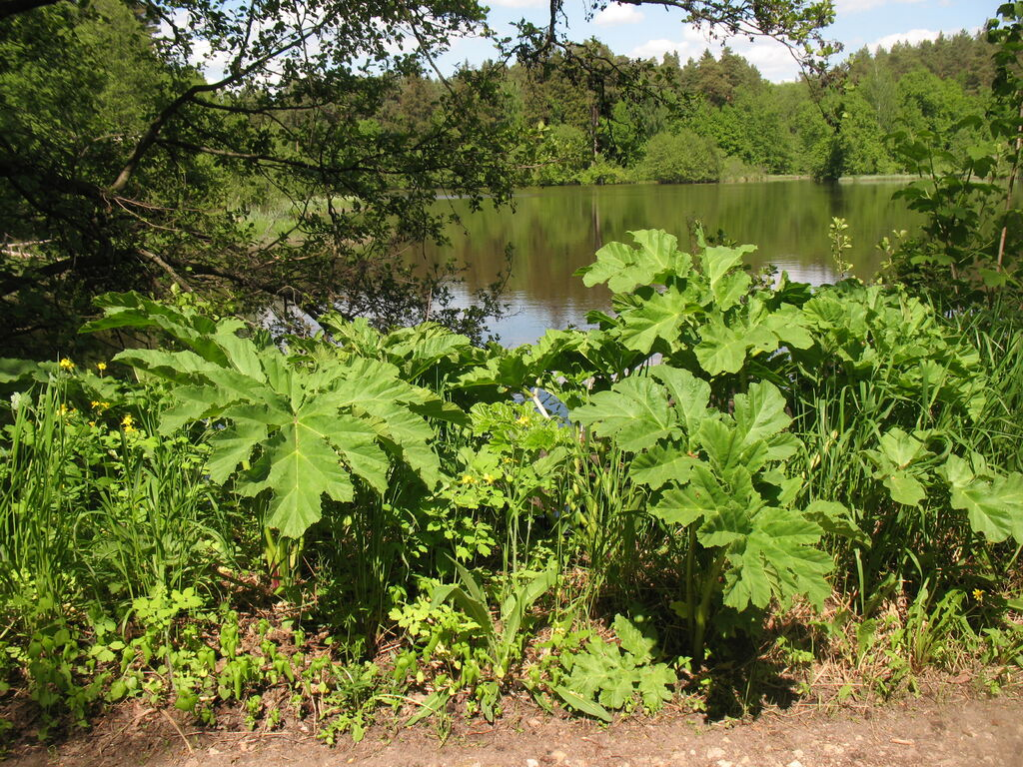
*Heracleumsosnowskyi* at the dam across the Tadenka River. Photo by Mikhail Shovkun.

Additionally, we counted the number of alien plant species for the Reserve. These were *Acer tataricum* L., *Acer negundo* L., *Malus prunifolia* (Willd.) Borkh., *Physocarpus opulifolius* (L.) Maxim., *Caragana arborescens* Lam., *Syringa vulgaris* L., *Solidago canadensis* L., *Solidago gigantea* Aiton, *Onobrychis arenaria* (Kit.) DC., *Allium ursinum* L., *Conyza canadensis* (L.) Cronquist, *Alkekengi officinarum* Moench, *Galega orientalis* Lam., *Heracleum sosnowskyi* Manden. (Fig. 3), *Aquilegia vulgaris* L., *Xanthoxalis fontana* (Bunge) Holub, *Torilis japonica* (Houtt.) DC., *Echinocystis lobata* (Michx.) Torr. & A.Gray and *Impatiens glandulifera* Royle. The statements about whether a species has been introduced to the territory of the Reserve were described according to the Establishment Means Controlled Vocabulary (see dwc: establishmentMeans in the dataset) and to the Degree of Establishment Controlled Vocabulary (see dwc: degreeOfEstablishment). Note, dwc: as degreeOfEstablishment is currently not supported, these data will be available later.

### Taxa included

**Table:** 

Rank	Scientific Name	
kingdom	Plantae	
kingdom	Fungi	
phylum	Tracheophyta	
phylum	Ascomycota	
phylum	Bryophyta	
phylum	Marchantiophyta	

## Temporal coverage

**Formation period:** July 27, 2019 - September 8, 2019; April 11, 2020 - August 30, 2020.

## Usage licence

### Usage licence

Other

### IP rights notes

Creative Commons Attribution (CC-BY) 4.0 License

## Data resources

### Data package title

Relevés of Main Vegetation Types of the Prioksko-Terrasnyi Biosphere Reserve (2019-2020)

### Resource link

https://www.gbif.org/dataset/bb6249ca-2e0b-449e-bd68-8d88bab4ed2b

### Alternative identifiers

http://gbif.ru:8080/ipt/resource?r=ptz_gb2020

### Number of data sets

1

### Data set 1.

#### Data set name

Vegetation Relevés of Main Vegetation Types of the Prioksko-Terrasnyi Biosphere Reserve (2019-2020)

#### Data format

Darwin Core Archive

#### Number of columns

52

#### Character set

UTF-8

#### 

**Data set 1.** 

Column label	Column description
eventID (Darwin Core Event, GBIF Relevé Extension, Darwin Core Occurrence Extension, MeasurementOrFact Extension)	An identifier for the relevé
rightsHolder (Darwin Core Event)	An organisation owning rights over the resource (Prioksko-Terrasnyi Biosphere Reserve)
sampleSizeValue (Darwin Core Event)	A numeric value for a measurement of the size of a sampling plot (100)
sampleSizeUnit (Darwin Core Event)	The unit of measurement of the size of a sampling plot (square metre)
samplingProtocol (Darwin Core Event)	The name of the method or protocol used during an Event (the relevé method, Braun-Blanquet cover-abundance scale)
eventDate (Darwin Core Event)	The date of an Event occurred (YYYY-MM-DD)
year (Darwin Core Event)	The four-digit year of the Event occurred
month (Darwin Core Event)	The integer month of the Event occurred
day (Darwin Core Event)	The integer day of the month of the Event occurred
habitat (Darwin Core Event)	A description of the habitat in which the Event occurred (in Russian)
eventRemarks (Darwin Core Event)	Comments or notes about the Event (in Russian)
country (Darwin Core Event)	The name of the country in which the Location occurs (Russian Federation)
countryCode (Darwin Core Event)	The standard code for the country where the Location occurs (RU)
stateProvince (Darwin Core Event)	The name of the next smaller administrative region than country in which the Location occurs (Moscow Region)
county (Darwin Core Event)	The name of the next smaller administrative region than stateProvince in which the Location occurs (Serpukhov district)
municipality (Darwin Core Event)	The of the next smaller administrative region than county in which the Location occurs (Danki)
locality (Darwin Core Event)	The specific description of the place (Prioksko-Terrasnyi Biosphere Reserve)
verbatimLatitude (Darwin Core Event)	The verbatim original latitude of the Location
verbatimLongitude (Darwin Core Event)	The verbatim original longitude of the Location
decimalLatitude (Darwin Core Event)	The geographic latitude of the Location in decimal degrees
decimalLongitude (Darwin Core Event)	The geographic longitude of the Location in decimal degrees
geodeticDatum (Darwin Core Event)	The spatial reference system (SRS) upon which the geographic coordinates given in decimalLatitude and decimalLongitude are based (WGS84)
coordinateUncertaintyInMeters (Darwin Core Event)	The horizontal distance (in metres) from the given decimalLatitude and decimalLongitude describing the smallest circle containing the whole of the Location.
coordinatePrecision (Darwin Core Event)	A decimal representation of the precision of the coordinates given in the decimalLatitude and decimalLongitude (0.00001)
language (Darwin Core Event)	A language of the resource (EN | RU)
coverTreesInPercentage (GBIF Relevé Extension)	The cover (%) of trees
coverShrubsInPercentage (GBIF Relevé Extension)	The cover (%) of shrubs
coverHerbsInPercentage (GBIF Relevé Extension)	The cover (%) of the herb layer
coverCryptogamsInPercentage (GBIF Relevé Extension)	The cover (%) of cryptogams
coverWaterInPercentage (GBIF Relevé Extension)	The cover (%) of open water
coverRockInPercentage (GBIF Relevé Extension)	The cover (%) of rocks
aspect (GBIF Relevé Extension)	The compass direction that the relevé site faces
inclinationInDegrees (GBIF Relevé Extension)	The angle of inclination of the relevé site in degrees, rounded to the nearest whole number
basisOfRecord (Darwin Core Occurrence Extension)	The specific nature of the data record (HumanObservation)
occurrenceID (Darwin Core Occurrence Extension)	An identifier for the Occurrence
recordedBy (Darwin Core Occurrence Extension)	A person responsible for recording the original Occurrence
occurrenceStatus (Darwin Core Occurrence Extension)	A statement about the presence or absence of a Taxon at a Location (present)
locationID (Darwin Core Occurrence Extension)	Vegetation layer code
locationRemarks (Darwin Core Occurrence Extension)	Vegetation layer description: A - the tree canopy layer , B - the understorey layer including tree undergrowth and tall shrubs, C - herbaceous layer together with tree and shrub seedlings, D - cryptogams layer
organismQuantity (Darwin Core Occurrence Extension)	Species abundance (r, +, 1, 2, 3, 4 or 5)
organismQuantityType (Darwin Core Occurrence Extension)	The type of quantification system used for the abundance (Braun-Blanquet scale)
identifiedBy (Darwin Core Occurrence Extension)	A person who assigned the Taxon to the occurrence
scientificName (Darwin Core Occurrence Extension)	The full scientific name of the Taxon
kingdom (Darwin Core Occurrence Extension)	The full scientific name of the kingdom in which the taxon is classified
phylum (Darwin Core Occurrence Extension)	phylum (Darwin Core Occurrence Extension)
class (Darwin Core Occurrence Extension)	The full scientific name of the class in which the taxon is classified
taxonRank (Darwin Core Occurrence Extension)	The taxonomic rank of the most specific name in the scientificName
establishmentMeans (Darwin Core Occurrence Extension)	Statement about whether an organism or organisms have been introduced to a given place and time through the direct or indirect activity of modern humans
degreeOfEstablishment (Darwin Core Occurrence Extension)	The degree to which an Organism survives, reproduces and expands its range at the given place and time
measurementType (MeasurementOrFact Extension)	The nature of the measurement, fact, characteristic or assertion (Forest type)
measurementValue (MeasurementOrFact Extension)	Forest type, see Table 1 for details
measurementMethod (MeasurementOrFact Extension)	A reference to (publication, URI) the method or protocol used to determine the forest type

## Figures and Tables

**Table 1.** Forest types distinguished in the dataset of relevés.

**Forest type**	**Code (dwc: measurementValue)**	**Number of relevés**
Nemoral-meadow herb aspen forest	MdNm_Aspen	2
Nemoral herb aspen forest	Nm_Aspen	8
Nemoral-boreal herb aspen forest	NmBr_Aspen	1
Nemoral and nitrophilous herb aspen forest	NmNt_Aspen	2
Piny-meadow herb aspen forest	PnMd_Aspen	1
Small boreal herb birch forest	Br_Birch	2
Hygrophytic birch forest	Hg_Birch	2
Meadow-nemoral herb birch forest	MdNm_Birch	5
Meadow and nitrophilous herb birch forest	MdNt_Birch	1
Nemoral herb birch forest	Nm_Birch	1
Nemoral and nitrophilous herb birch forest	NmNt_Birch	2
Nitrophilous herb birch forest	Nt_Birch	1
Oligotrophic herb-sphagnum birch forest	Olg_Birch	3
Piny-meadow herb birch forest	PnMd_Birch	1
Nemoral and nitrophilous herb black alder forest	NmNt_BlAlder	3
Nitrophilous herb black alder forest	Nt_BlAlder	8
Meadow-nemoral herb deciduous forest	MdNm_Decds	7
Nemoral-boreal herb deciduous forest	NmBr_Decds	1
Nemoral and nitrophilous herb deciduous forest	NmNt_Decds	2
Meadow-nemora herb linden forest	MdNm_Linden	2
Nemoral herb linden forest	Nm_Linden	10
Nemoral-boreal herb linden forest	NmBr_Linden	1
Nemoral and nitrophilous herb linden forest	NmNt_Linden	2
Meadow-nemoral herb oak forest	MdNm_Oak	2
Nemoral herb oak forest	Nm_Oak	9
Piny-meadow herb oak forest	PnMd_Oak	1
Small boreal herb pine forest	Br_Pine	3
Meadow herb pine forest	Md_Pine	2
Meadow-nemoral herb pine forest	MdNm_Pine	8
Nemoral herb pine forest	Nm_Pine	9
Nemoral-boreal herb pine forest	NmBr_Pine	9
Oligotrophic herb-sphagnum pine forest	Olg_Pine	6
Piny herb pine forest	Pn_Pine	4
Piny-boreal herb pine forest	PnBr_Pine	6
Piny-meadow herb pine forest	PnMd_Pine	7
Pine forest without herbaceous layer	None_Pine	1
Boreal-nemoral herb spruce forest	BrNm_Spruce	12
Boreal and nitrophilous herb spruce forest	BrNt_Spruce	1
Boreal and nitrophilous herb spruce forest developed after bark beetle	BrNt_SpruceABB	27
Nemoral and nitrophilous herb spruce forest	NmNt_Spruce	2
Hygrophytic willow bush	Hg_Willow	3
Nemoral and nitrophilous herb willow forest	NmNt_Willow	3
Meadow herb glade	Md_Glade	35
Meadow and nitrophilous herb glade	MdNt_Glade	4
Piny herb glade	Pn_Glade	1
Piny-meadow herb glade	PnMd_Glade	9
Riparian	Riparian community	4
Hygrophytic meadow	Hg_Md	3
Mesophytic meadow	Meso_Md	18
Oligotrophic and mesotrophic bog	Olg_Bog	12
